# Results of a pilot study to improve identification and referral for people at high risk for hereditary colorectal cancer syndromes

**DOI:** 10.1186/1897-4287-9-S1-P21

**Published:** 2011-03-10

**Authors:** Anna Leininger, Kristin B Niendorf, Timothy Church, Robert D Madoff

**Affiliations:** 1 William C. Bernstein MD Familial Cancer Registry, University of Minnesota, MN 55455, USA; 2 Division of Environmental Health Sciences, University of Minnesota, Minneapolis, MN 55455, USA; 3 Division of Colon and Rectal Surgery, University of Minnesota, Minneapolis, MN 55455, USA

## Background

Five percent of colorectal cancers are attributable to hereditary syndromes for which genetic testing is clinically available. Effective surveillance strategies and risk-reducing surgeries lower risk for people diagnosed with inherited colorectal cancer syndromes. However, from a public health standpoint, the existence of effective interventions is futile if not applied broadly and appropriately to the population at risk. Efficient identification of people with hereditary risk remains a challenge, and failure to systematically identify people at high hereditary risk results in preventable morbidity and mortality. The William C. Bernstein M.D. Familial Cancer Registry (Bernstein Registry) is a research-based program that triages participants for hereditary cancer risk. Self-reported family histories are assessed and participants are given tailored information about their hereditary risk factors. People at high risk are given information about the availability and advisability of clinical genetics services. Pilot populations are being enrolled to establish registry processes and determine the feasibility of implementation at a population-based level. This pilot group includes volunteers from a previously established colorectal cancer registry.

## Materials and methods

A 7-item screening questionnaire determines potential eligibility for the Bernstein Registry using elements of early onset, multiple primary and family history of cancers. Potentially eligible participants are contacted by telephone and a four-generation cancer history is generated via guided interview. Pedigrees are analyzed by genetic counselors for hereditary cancer risk probabilities based on published guidelines. Targeted, tailored risk information is provided in writing along with a copy of the family tree. A list of genetic counseling service providers is included for people at high risk.

## Results

Four hundred sixty six (466) of 898 people (52%) in the Minnesota Colorectal Cancer Initiative (MCCI) registry have histories eligible for the Bernstein registry. To date, 437 invitations have been mailed to this group, 55% responded. Of 242 completed eligibility screeners, 239 proved eligible (99%), and 155 (64%) have enrolled. Of 55 completed assessments, 44 have been classified as high risk, with genetic counseling/testing recommended.

## Conclusions

The Bernstein Registry can systematically determine who may benefit from clinical genetics services (genetic counseling and testing). A majority of individuals from families previously identified as high risk are motivated to participate in research and tolerate the relatively onerous enrollment process. Whether this same population will follow through on recommendations to pursue genetic counseling and testing, and whether the intensive risk-identification process is feasible and sustainable on a public health level is the target of future research.

